# Prevalence and temporal changes of mutations linked to antimalarial drug resistance in *Plasmodium falciparum* and *Plasmodium vivax* in Palawan, Philippines

**DOI:** 10.1016/j.ijid.2021.12.318

**Published:** 2022-03

**Authors:** Alison Paolo N. Bareng, Lynn Grignard, Ralph Reyes, Kim Fornace, Freya Spencer, Ma. Lourdes Macalinao, Jennifer Luchavez, Fe Esperanza Espino, Chris Drakeley, Julius Clemence R. Hafalla

**Affiliations:** a Department of Parasitology and National Reference Centre for Malaria and Other Parasites, Research Institute for Tropical Medicine, Department of Health, Muntinlupa City, Philippines; b Department of Infection Biology, Faculty of Infectious and Tropical Diseases, London School of Hygiene and Tropical Medicine, London, United Kingdom

**Keywords:** CQ, chloroquine, SP, sulphadoxine/pyrimethamine, AL, artemisinin + lumefantrine, WHO, World Health Organisation, DHFR, dihydrofolate reductase, DHPS, dihydropteroate synthase, CRT, CQ resistance transporter, k13, kelch-13 protein, MDR1, multidrug-resistance 1, Pf, *Plasmodium falciparum*, Pv, *Plasmodium vivax*, DNA, deoxyribonucleic acid, PCR, Polymerase chain reaction, TES, Therapeutic Efficacy Surveillance, rRNA, ribosomal ribonucleic acid, Malaria, *Plasmodium*, Drug resistance, Single nucleotide polymorphism, Mutation, Elimination

## Abstract

**Objective:**

This study provides 2016 data on the prevalence of key single nucleotide polymorphisms (SNPs) associated with antimalarial drug resistance in Palawan, Philippines. Findings were combined with historical data to model temporal changes in the prevalence of these SNPs in *Plasmodium* isolates.

**Methods:**

*Plasmodium* isolates were genotyped using drug resistance markers *pfmdr1, pfcrt, pfdhfr, pfdhps*, kelch-13, *pvmdr1, pvdhfr*, and *pvdhps*. Temporal trends in the probability of mutations were estimated as a function of time using a binomial generalised linear model.

**Results:**

All samples sequenced for *Plasmodium falciparum* chloroquine markers *pfmdr1* and *pfcrt* had wild-type alleles. Varying mutation patterns were observed for the sulphadoxine/pyrimethamine markers *pfdhps* and *pfdhfr*; complete quintuplet mutations were not found. No SNPs were observed for the artemisinin marker kelch-13. For *Plasmodium vivax*, differing patterns were detected for *pvmdr1, pvdhfr*, and *pvdhps*.

**Conclusions:**

The study findings suggest that the current drugs remain effective and that there is limited importation and establishment of resistant parasites in the area. Clear temporal trends were recognised, with prominent decreases in the proportions of *pfcrt* and *pfmdr* mutations detected within the past 15 years, consistent with a change in antimalarial drug policy. Continuous surveillance of antimalarial drug resistance is important to support malaria elimination efforts.

## Highlights

• *Plasmodium falciparum* and *Plasmodium vivax* isolates from the Philippines were analysed.
• Varying mutations were found in markers linked to resistance to antimalarial drugs.
• None of the mutations were particularly of high prevalence.
• Clear temporal patterns in these mutations were observed within the past 15 years.
• Decrease in *pfcrt* and *pfmdr* mutations are in line with antimalarial policy change.

## Introduction

Malaria remains one of the world's greatest public health challenges. In 2019, it was responsible for nearly 229 million cases and 409 000 deaths worldwide. Whilst 800 000 people remain at high risk of contracting malaria in the Philippines (World Health Organization, 2020), only three provinces reported indigenous cases in 2020; the province of Palawan reported 97% of all the cases in the country (Maru, 2020). Despite current innovative strategies to accelerate global control and elimination, including in the Philippines, with a huge decline in global malaria cases and deaths seen over the past years (World Health Organization, 2020), the emergence and spread of antimalarial drug resistance threatens effective treatment in endemic countries (Menard and Dondorp, 2017). Resistance to commonly used antimalarials has become widespread in low transmission settings, particularly in Southeast Asia (Baird, 2009; Baird et al., 1991; Eyles et al., 1963; Guthmann et al., 2008; Hurwitz et al., 1981; Ratcliff et al., 2007; Young et al., 1963), threatening elimination efforts and compelling countries to modify their treatment strategies and policies.

There is a commitment from the Government of the Philippines to eliminate malaria by 2030 together with the rest of the Asia-Pacific region (Department of Health, 2019, Wen et al., 2016; ). Since the late 1990s, the Philippines has monitored the efficacy of first-line antimalarial drugs for *Plasmodium falciparum* following the World Health Organization (WHO) protocol for Therapeutic Efficacy Surveillance (TES) (UCSF Global Health Group, 2014). Although drug resistance genotyping of TES isolates has been limited, results led to revisions of the National Malaria Control Programme treatment guidelines for falciparum malaria in 2002, from the use of chloroquine (CQ) alone to CQ plus sulphadoxine/pyrimethamine (SP) combination (UCSF Global Health Group, 2014). Due to observed unacceptable levels of treatment failure to the triple drug combination, the National Malaria Control Programme in 2009 adopted artemisinin + lumefantrine (AL) treatment for *P. falciparum* (DOH, 2020a). Based on WHO guidelines (World Health Organization, 2015), the Philippines revised its *Plasmodium vivax* treatment policy from CQ to AL in 2018.

Correlating the data from available clinical studies with genetic markers relating to drug resistance and tracking temporal changes in these markers could be valuable for guiding national malaria treatment policies (Djimdé et al., 2001; Mugittu et al., 2004). SP resistance in *P. falciparum* is linked to key mutations in dihydrofolate reductase (DHFR) N_51_, C_59_, S_108_ and dihydropteroate synthase (DHPS) S_436_, A_437_, K_540_, A_581_, A_613_ (Kublin et al., 2002; Staedke et al., 2004), whilst mutations in CQ resistance transporter (CRT) C_72_, V_73_, M_74_, N_75_, K_76_ and multidrug-resistance 1 (MDR1) N_86_, Y_184_ are associated with CQ resistance in *P. falciparum* (Babiker et al., 2001; Das et al., 2014; Fidock et al., 2000). For *P. vivax*, the role of mutations in the MDR1 orthologue in CQ (Y_976_, F_1076_) resistance remains unresolved due to issues with relapse and recrudescence (Price et al., 2012), whilst a panel of mutations in the DHFR (I_13_, P_33_, F_57_, S_58_, T_61_, S_117_, I_173_) (Imwong et al., 2003) and DHPS (S_382_, A_383_, K_512_, A_553_, V_585_) orthologues are associated with an altered clinical response to SP (Hawkins et al., 2007)*.* Additionally, recent studies have suggested that the delay in *P. falciparum* clearance with the current first-line drug, AL, is linked to mutations in the gene encoding the propeller region of the kelch-13 protein (k13) (Menard, 2016). A combination of clinical AL resistance and k13 mutations has been observed in Southeast Asian countries (Menard, 2016); no confirmation is available from the Philippines.

In order to provide recent data for the Philippines, this study presents findings on the prevalence of mutations based on the genetic markers *pfmdr1, pfcrt, pfdhfr, pfdhps, pf*k13, *pvmdr1, pvdhfr*, and *pvdhps* from samples collected in the Philippines as part of a research study to employ enhanced surveillance methodologies in the country (Reyes et al., 2021). Furthermore, modelling outcomes on temporal trends in the prevalence of these mutations after combining results with historical data are also presented.

## Materials and methods

### Study population and sample collection

**Figure 1:**
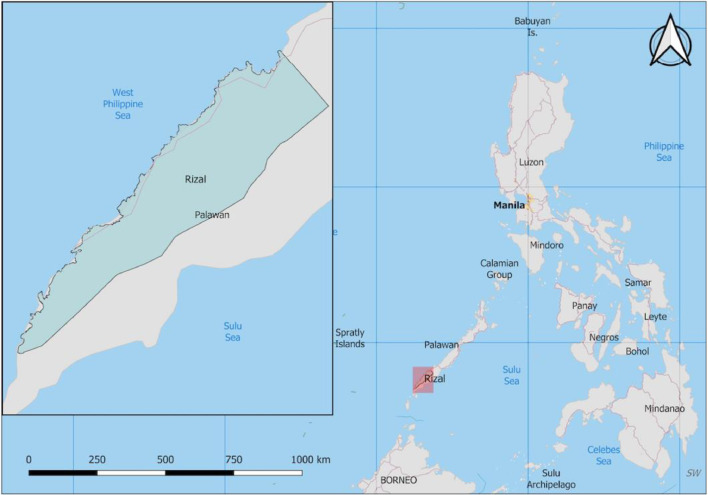
Location of the study site. Map highlighting the Municipality of Rizal in the island province of Palawan, Philippines (inset: closer view of the Municipality of Rizal).

Samples were collected between June and December 2016 in the Municipality of Rizal in the southwest of Palawan Province in the Philippines, as part of a larger survey (Reyes et al., 2021; Figure 1; **Supplementary Material** Table S1). Palawan is a malaria endemic province, with over 7000 cases reported in 2015 (DOH, 2020b). Rizal municipality recorded the highest number of infections out of a population of approximately 50 000 (PSA, 2020).

Study participants were (1) outpatients of all ages who consulted the rural health unit regardless of symptoms, and (2) the patient's companion. Both the patient and the companion had been residents of the area for at least 7 days prior to the rural health unit visit. Depending on their age group, potential participants were asked to sign an informed consent and/or an assent form. Patients with a serious illness who required urgent care or transportation to a higher-level health facility were excluded. Blood from a finger prick was collected from each participant for microscopy examination of malaria blood film, rapid diagnostic tests, and preparation of dried blood spots for molecular assays. Details of the study design and sampling have been reported previously (Reyes et al., 2021).

### DNA extraction and confirmation of *Plasmodium sp* through nested PCR

Parasite DNA was extracted from filter blood spots using a QIAamp DNA Mini Kit (Qiagen, Germany) with a final elution at 100 μl, or by Chelex extraction method (Bio-Rad Laboratories, Hercules, CA, USA) with a final volume of 180 μl. Two to three blood spots, each with an approximate volume of 20 μl, were used. Both protocols were performed in accordance with the manufacturer's instructions. The extracted DNA samples were stored at −20°C until use. Genus and species-specific *Plasmodium* DNA in the extracted samples were confirmed using a modified standard nested-PCR assay, which targeted a highly conserved region of the 18s rRNA gene (Fuehrer et al., 2011; Singh and Snounou, 2002; Snounou et al., 1993). The results of the PCR amplifications have been published elsewhere (Reyes et al., 2021).

### Amplification and sequencing of *P. falciparum* and *P. vivax* drug resistance markers

The prevalence of genetic polymorphisms associated with antimalarial resistance was determined using nested-PCR protocols. Sequences of *P. falciparum mdr1, crt, dhfr, dhps*, and k13 molecular markers were amplified following protocols performed in published studies (Abdoulaye et al., 2001, Ariey et al., 2014; Humphreys et al., 2007; Lo et al., 2013) . Genetic markers of *P. vivax mdr1, dhfr*, and *dhps* were also amplified using published methods (Lekweiry et al., 2012). The numbers of successful amplifications are given in the Results section.

The amplified targets were purified using a QIAquick PCR Purification Kit (Qiagen, Germany). Sequencing reactions were performed using the Big Dye Terminator Cycle Sequencing Kit (Applied Biosystems) following the manufacturer's instructions. The reactions involved the use of the forward and reverse primers of the second round PCR.

### Sequence data analysis

Gene sequences were aligned and analysed using CLC Sequence Viewer (version 8). The reference standards were retrieved from PlasmoDB: *pfmdr1-* (**PF3D7_0523000**), *pfcrt*- **(PF3D7_0709000**), *pfdhfr*- (**PF3D7_0417200**), *pfdhps*- (**PF3D7_0810800**), k13- (**PF3D7_1343700**), *pvmdr1*- (**PVX_080100**), *pvdhfr*- (**PVX_089950**), *pvdhps*- (**PVX_123230**).

### Modelling of temporal trends in malaria drug resistance markers

Historical data were collated from the literature (Auliff et al., 2006; Bareng et al., 2018; Chen et al., 2005, Chen et al., 2003; Hatabu et al., 2009; Macalinao et al., 2019; Menard, 2016; Saito-Nakano et al., 2008; Sakihama et al., 2007) and unpublished data from (Segubre-Mercado et al., n.d.) and (Bareng et al., n.d.) The compiled data are summarised in **Supplementary Material** Tables S2 and S3. Along with the data obtained from the current study, the probability of detecting specific mutations was estimated during each time point as a binomial distribution of *p* mutations detected from *n* samples screened. For mutations with over two time points, temporal trends in the probability of mutations as a function of time were estimated using a binomial generalised linear model. Analyses were completed and visualised in R statistical software (v. 3.6).

## Results

### Analysis of single nucleotide polymorphisms (SNPs) in *P. falciparum* resistance markers

**Figure 2:**
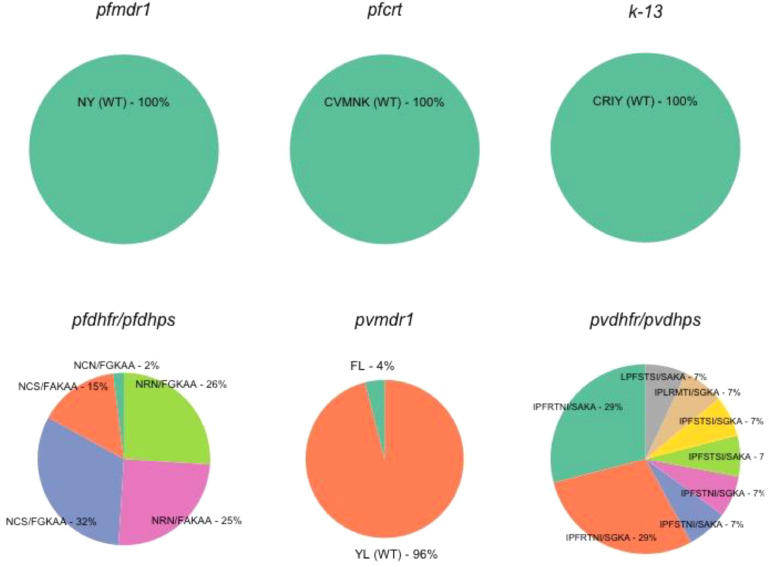
Percentages of haplotypes in drug resistance markers *pfmdr1, pfcrt*, k13, *pvmdr1*, and combination alleles *dhfr–dhps* in *Plasmodium* isolates.

*mdr1* and *crt*, genes associated with CQ resistance in *P. falciparum*, were successfully amplified and sequenced in 90 and 51 samples, respectively. The amino acid variants and their distribution are summarised in Table 1 and Figure 2. Notably, the wild-type genes corresponding to N_86_Y_186_ for *pfmdr1* and C_72_V_73_M_74_N_75_K_76_ for *pfcrt* were found in all of the samples sequenced.

**Table 1:** Distribution of amino acid variants in the genetic markers conferring antimalarial drug resistance in *Plasmodium falciparum* and *Plasmodium vivax*

Genetic markers (Number of genotyped samples)	Amino acid position	Amino acid^a^	Frequency (%)
*pfdhfr* (*n* = 123)	51	N (WT)	123 (100%)
	59	C (WT)	59 (48%)
		R (Mut)	64 (52%)
	108	S (WT)	57 (46%)
		N (Mut)	66 (54%)
*pfdhps* (*n* = 64)	436	F (Mut)	64 (100%)
	437	A (WT)	24 (38%)
		G (Mut)	40 (63%)
	540	K (WT)	63 (98%)
		E (Mut)	1 (2%)
	581	A (WT)	64 (100%)
	613	A (WT)	64 (100%)
*pvmdr1* (*n* = 27)	976	Y (WT)	26 (96%)
		F (Mut)	1 (4%)
	1076	L (Mut)	27 (100%)
*pvdhfr* (*n* = 25)	13	I (WT)	24 (96%)
		L (Mut)	1 (4%)
	33	P (WT)	25 (100%)
	57	F (WT)	24 (96%)
		L (Mut)	1 (4%)
	58	S (WT)	10 (40%)
		R (Mut)	15 (60%)
	61	T (WT)	24 (96%)
		M (Mut)	1 (4%)
	117	S (WT)	9 (36%)
		N (Mut)	15 (60%)
		T (Mut)	1 (4%)
	173	I (WT)	25 (100%)
*pvdhps* (*n* = 18)	382	S (WT)	18 (100%)
	383	A (WT)	11 (61%)
		G (Mut)	7 (39%)
	512	K (WT)	18 (100%)
	553	A (WT)	17 (94%)
		T (Mut)	1 (6%)

Genetic markers that do not have mutations are not included in the table.

a WT = wild-type allele; Mut = mutant allele.

*dhfr*, one of the genes associated with SP resistance, was successfully sequenced in 123 samples. Forty-six percent (*n* = 57) of the isolates sequenced had the wild-type sequence (N_51_C_59_S_108_), whilst the majority (52%, *n* = 64) of the products had a double mutation at positions N_51_R_59_N_108_, whilst two samples yielded a single mutation at position N_51_C_59_N_108_. For *dhps*, a gene also associated with SP resistance, 64 of 165 samples were successfully sequenced. No *dhps* wild-type isolates were identified. Isolates carrying double mutations F_436_G_437_K_540_A_581_A_613_ were found in 61% (*n* = 39) of the samples. Single mutation F_436_A_437_K_540_A_581_A_613_ was also present in 24 samples (37%), and one isolate (2%) carried a triple mutant at position F_436_G_437_E_540_A_581_A_613_. The proportion of combination alleles *dhfr–dhps* in *P. falciparum* species was investigated (Figure 2). The most prevalent combination in *P. falciparum* carried a wild-type *dhfr* and double mutation *dhps* (32%), followed by the double mutant *dhfr* combined with double mutant *dhps* (26%). Whilst no wild types were detected for both *pfdhfr* and *pfdhps* haplotypes, the full set of quintuplet mutations highly associated with SP treatment failure was not found in this study. Regarding the k13 markers, all sequenced samples (*n* = 57, 100%) yielded wild-type alleles for codons that are known to be associated with AL susceptibility.

The design of the original study (Reyes et al., 2021) from which these samples were obtained included all health facility attendees, regardless of symptoms. A preliminary analysis was performed to compare the mutations amongst subclinical (participants who presented without fever) and symptomatic (with fever) infections. This analysis revealed that the frequencies of haplotypes with mutation(s) were comparable between the two groups (**Supplementary Material** Table S4).

### Analysis of SNPs in *P. vivax* genetic markers

Of the 57 confirmed *P. vivax* samples, genetic markers *pvmdr1, pvdhfr*, and *pvdhps* were successfully amplified and sequenced for 27, 25, and 18 samples, respectively, using nested PCR. A summary of the observed allelic distribution of *P. vivax* markers is given in Table 1 and shown in Figure 2 . Analysis of the *pvmdr1* samples showed that the majority (*n* = 26, 96%) had a single mutation at position Y_956_L_1076_, whilst only one sample (*n* = 1, 4%) carried double mutations F_976_L_1076_. No *pvmdr1* wild-type isolates were identified. For *pvdhfr*, wild-type alleles were detected in 32% (*n* = 8) of the samples. Samples carrying the double mutation at positions I_13_P_33_F_57_R_58_T_61_N_117_I_173_ had the highest prevalence (56%, *n* = 14). For the observed *pvdhps* loci, the haplotype S_382_A_383_K_512_A_553_ dominated most of the samples with 61% (*n* = 11), followed by single mutation at position S_382_G_383_K_512_A_553_ with 33% (*n* = 6). The prevailing haplotypes with equal numbers of frequencies were double mutation *dhfr* combined with single mutant *dhps* and double mutant *dhfr* and no resistant mutant *dhps* (29% each) (Figure 2). Given the limited *P. vivax* isolates in this study, the mutant alleles for *dhfr* were found to be more frequent in subclinical infections (**Supplementary Material** Table S4).

### Temporal trends in drug resistance markers in the Philippines

**Figure 3:**
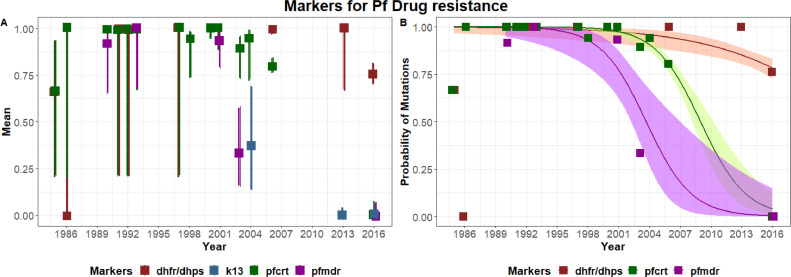
Temporal trends in the probability of mutations for *Plasmodium falciparum* isolates in the Philippines. (A) Mean probability of mutation and 95% confidence intervals for each sampled time point. (B) Modelled probability of detecting specific mutations over time and associated 95% confidence intervals. Data were assembled from this study and historical findings (**Supplementary Material** Table S2).

**Figure 4:**
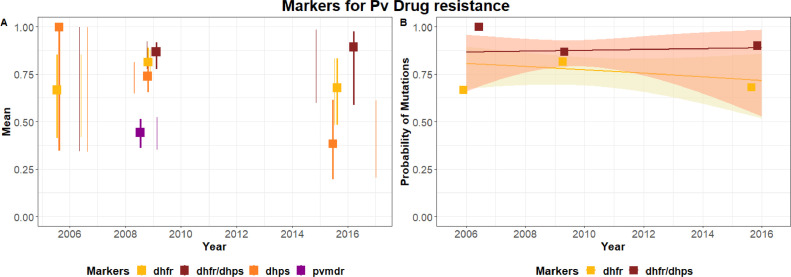
Temporal trends in the probability of mutations for *Plasmodium vivax* isolates in the Philippines. (A) Mean probability of mutation and 95% confidence intervals for each sampled time point. (B) Modelled probability of detecting specific mutations over time and associated 95% confidence intervals. Data were assembled from this study and historical findings (**Supplementary Material** Table S3).

To model temporal changes in the prevalence of mutations linked to antimalarial drug resistance in both *P. falciparum* and *P. vivax* from different areas in the Philippines, historical and current data were assembled. Data included *P. falciparum* and *P. vivax* samples collected as far back as 1985 and 2002, respectively (Auliff et al., 2006; Bareng et al., 2018; Chen et al., 2005, Chen et al., 2003, Hatabu et al., 2009; Macalinao et al., 2019; Menard, 2016; Saito-Nakano et al., 2008; Sakihama et al., 2007). The mean probabilities of detecting mutations and associated 95% confidence intervals for each species are presented in Figure 3 (for *P. falciparum*) and Figure 4 (for *P. vivax*). Low sample sizes in the numbers of samples screened historically resulted in wide confidence intervals. For the mutations with sufficient data at multiple time points, clear temporal trends were identified, with marked reductions in the proportions of *pfcrt* and *pfmdr* mutations detected within the past 15 years (Figure 3). In contrast, only limited changes were detected in *dhfr* and *dhps* mutations in both malaria species, with high probabilities of detecting these mutations at recent time points.

## Discussion

This aim of this study was to provide up to date evidence on drug resistance through the analysis of SNP markers for common antimalarial drugs in one of most endemic municipalities in the Philippines. Furthermore, given historical data on genetic markers of antimalarial drug resistance in the Philippines, this study offers an analysis of changes over time in these markers since sequencing data became available.

In this study, a low prevalence of mutations was found for drugs for both *P. falciparum* and *P. vivax* . Notably, the prevalence of mutations in k13, which is associated with resistance to the first-line drug AL, was 0%. Specifically, low-grade genetic modifications were observed for drug resistance markers for *P. falciparu*m. Results obtained for SP resistance markers were consistent with those of previous studies from the Philippines that were conducted in Cordillera Administrative Region, Davao del Sur, Sultan Kudarat, Zamboanga City, and Palawan (*n* = 310) in 2003–2007 (Segubre-Mercado et al., n.d.) and in CARAGA region (*n* = 38) in 2005–2006 (Macalinao et al., 2019) . Similar results were also seen in some other Southeast Asian countries (Biswas et al., 2000; Khim et al., 2005). However, in the present study, the majority of the samples had the F_436_ mutation in the *dhps* marker, while in the investigation done by Segubre et al. (Segubre-Mercado et al., n.d.), all samples yielded the wild-type at this position. It should be noted, however, that the detected SNPs may not indicate high-level resistance, as several studies have suggested that quintuple mutation of the combination haplotype *pfdhfr* and *pfdhps* is highly likely to be associated with SP treatment failure (Kublin et al., 2002; Picot et al., 2009; Staedke et al., 2004). In contrast, mutations were completely absent in genetic markers associated with CQ resistance *pfcrt* and *pfmdr1*. Interestingly, earlier studies with samples collected in 1984–2007 in different locations in the Philippines (Chen et al., 2005; Chen et al., 2003, Hatabu et al., 2009; Macalinao et al., 2019; Saito-Nakano et al., 2008; Sakihama et al., 2007) reported either single C_72_T_76_ or double S_72_T_76_ mutations in the *pfcrt* marker. The observed absence of mutation(s) in the molecular markers linked with CQ resistance in this study further supports the reversion of resistance genes in the absence of ongoing drug pressure (Kublin et al., 2003; Mang'era et al., 2012; Mohammed et al., 2013; Pelleau et al., 2015) and presents the possibility of reassessing the therapeutic efficacy of earlier generations of antimalarials. Lastly, the k13 marker was also investigated in this study and it was found that none of the isolates had SNPs at known Asian resistance-conferring alleles: C_580_Y, R_539_T, I_543_T, and Y_493_H (Menard, 2016). Moreover, no mutations were observed in Philippines samples collected in 2013 (Menard, 2016). Continuous molecular surveillance on the efficacy of these drugs, in addition to clinical studies and in vitro responses, could shed light on the significance of these mutations.

For *P. vivax* infections, high frequencies of single mutation T_976_L_1076_ (96.00%) in the *pvmdr1* gene were detected in this study, corroborating the results from previous TES samples collected in Palawan in the years 2009–2012 (Bareng et al., n.d.) and in some Asian countries (Imwong et al., 2008; Kim et al., 2011; Lin et al., 2013; Lu et al., 2011). However, given that CQ is still effective against *P. vivax* infections, this finding may further provide evidence that this mutation is just a geographical variant and does not indicate CQ resistance (Lekweiry et al., 2012). Moreover, *pvdhfr* genotyped samples mostly had mutations in only two out of seven alleles, whilst *pvdhps* sequenced samples were mostly wild types. The results were identical to those of a previous study on samples collected in Palawan (Bareng et al., 2018) and Agusan del Sur (Auliff et al., 2006). These results may provide a further indication of the sensitivity of *P. vivax* to available treatments. Nevertheless, additional investigations are needed to ascertain whether the observed mutations reflect drug responsiveness or merely another case of geographical variation as described in another study (Lekweiry et al., 2012).

Temporal trends in the mutations linked to antimalarial drug resistance were modelled using historical data and those generated in this study. The historical data included data from all over the country and not only from Rizal, Palawan. As mutations screened at different time points were not designed to be population representative, the modelling results cannot be used to generalise about the prevalence of mutations in wider parasite populations. Yet, the clear temporal trends in the numbers of mutations detected at different sampling points suggests wider changes in the frequency of mutations over time. Nevertheless, the notable decline in the proportions of *crt* and *mdr* mutations detected within the past 15 years (prior to the 2016 collection of the samples for this study) is in line with the country's change in antimalarial drug policy in 2009, when the Philippines took up AL treatment for *P. falciparum* due to response failures to CQ and SP (DOH, 2020a).

It has been suggested that the persistence of some key mutations following years of drug cessation could be attributed to the subclinical and asymptomatic infections harbouring these polymorphisms (Brown et al., 2012; Dokunmu et al., 2019; Nyunt et al., 2017). These infections could cause the continued transmission of parasites harbouring polymorphisms in the population even after an intervention. It is noteworthy that in the current study, participants with subclinical infections were not followed up to determine whether the infections remained subclinical (asymptomatic) or progressed to symptomatic disease. Besides the mutant alleles for *P. vivax dhfr*, initial analysis showed that participants with subclinical and symptomatic infections harbour parasites with comparable frequencies of haplotypes with mutation(s).

The integration of molecular techniques, such as drug resistance genotyping, with current malaria surveillance methods would be useful to the malaria programme, as it has the potential to allow the emergence of resistant parasites to be monitored continuously in an area . This enhanced surveillance is in line with control and elimination efforts, including the possible widespread use of drugs for mass administration. This study could also be useful in assisting the authorities in implementing the country's malaria treatment policies properly and suggests that population-based drug delivery would not be affected by drug resistance. Further work could also be done to analyse within-host diversity in the parasites, which would identify minor clones that may harbour genes conferring drug resistance.

In conclusion, varying mutation patterns were seen in different genetic markers associated with antimalarial drug resistance, but none at particularly high prevalence. Mutations were still present in SP markers *pfdhfr* and *pfdhps*; similar observations were seen with TES samples collected in 2001. In contrast, the observed alleles in genetic markers linked with CQ resistance in *P. falciparum* have all turned into wild-type genotypes after 15 years; these possibly reverted back to the original gene structure. SNPs associated with *P. vivax* resistance were still identified in both CQ and SP markers, similar to what was observed in TES 2009 samples. The roles of these mutations require interpretation alongside clinical and in vivo studies. Consequently, given the regional concerns around resistance, further monitoring of drug resistance using molecular and clinical responses seems worthwhile, as part of control and elimination efforts. In summary, the data and results of this study indicate that (1) the current first-line treatments should be effective, and (2) there has been relatively little importation and successful establishment of parasites from areas where resistance and resistance markers are more established.

## WEB REFERENCES

Department of Health (DOH), Republic of the Philippines, Administrative order No. 2009-0001: “Revised policy and guidelines on the diagnosis and treatment of malaria”. DOH website (2020) [https://dmas.doh.gov.ph:8083/Rest/GetFile?id=336782]. Date (Accessed: 09 November 2020).

Department of Health (DOH), Republic of the Philippines, Statistics: Distribution of cases in the Philippines (2020) [https://doh.gov.ph/malaria-control-program]. Date (Accessed: 09 November 2020).

Department of Health (DOH), Republic of the Philippines, PHL Malaria-free by 2030. DOH website (2019). [https://doh.gov.ph/press-release/Phl-Malaria-Free-by-2030-DOH] (Accessed: 24 September 2021)

Maru D. Philippines on track to eradicate malaria by 2030-WHO. ABS-CBN News. [https://news.abs-cbn.com/news/02/13/20/philippines-on-track-to-eradicate-malaria-by-2030-who] (Accessed: 24 September 2021).

Philippine Statistics Authority (PSA), Highlights of Philippine Population 2015 Census of Population, R04B (2020) [https://psa.gov.ph/population-and-housing/statistical-tables/title/Highlights%20of%20the%20Philippine%20Population%20%202015%20Census%20of%20Population]. Date (Accessed: 09 November 2020).

## Appendix Supplementary materials

Image, application 1
