# Supplementary material for: Prevalence and temporal changes of mutations linked to antimalarial drug resistance in Plasmodium falciparum and Plasmodium vivax in Palawan, Philippines
Source: Int J Infect Dis. 2022 Mar;116:174–81. doi: 10.1016/j.ijid.2021.12.318 (PMC8866131; doi:10.1016/j.ijid.2021.12.318)
Supplement: Supplementary file 1 [file mmc1.docx]

| **Supplementary Table 1.** | | |
| --- | --- | --- |
| Summary of samples used in this study | | |
| Species PCR breakdown | Pf | 142 |
|  | Pv | 37 |
|  | Mixed | 23 |
|  | Total | **202** |
| Fever? | Yes | 88 |
|  | No | 114 |

| **Supplementary Table 2**. Data^a^ used for modelling temporal trends in *P. falciparum* mutations | | | | | | |
| --- | --- | --- | --- | --- | --- | --- |
| **Genetic marker  (n of genotyped samples)** | **Study site** | **Date of collection** | **Amino acid position** | **Amino acid** | **Frequency (%)** | **Reference** |
| pfcrt (n=9) | Imported cases from the Philippines (Locations unspecified) | 1985-1998 | 72 | C (WT) | 3 (33%) | Saito-Nakano et al., 2008 |
|  |  |  |  | S (Mut) | 6 (67%) |  |
|  |  |  | 73 | V (WT) | 9 (100%) |  |
|  |  |  | 74 | M (WT) | 8 (89%) |  |
|  |  |  |  | I (Mut) | 1 (11%) |  |
|  |  |  | 75 | N (WT) | 8 (89%) |  |
|  |  |  |  | E(Mut) | 1 (11%) |  |
|  |  |  | 76 | K (WT) | 2 (22%) |  |
|  |  |  |  | T (Mut) | 7 (78%) |  |
| pfdhfr (n=7) | Imported cases from the Philippines (Locations unspecified) | 1985-1998 | 51 | N (WT) | 7 (100%) | Saito-Nakano et al., 2008 |
|  |  |  | 59 | C (WT) | 3 (43%) |  |
|  |  |  |  | R (Mut) | 4 (57%) |  |
|  |  |  | 108 | S (WT) | 2 (29%) |  |
|  |  |  |  | N (Mut) | 5 (71%) |  |
| pfcrt (n=12) | Morong, Bataan, Luzon | 1989-1991 | 72 | C (WT) | 10 (83%) | Chen et al., 2003 |
|  |  |  |  | S (Mut) | 2 (17%) |  |
|  |  |  | 74 | M (WT) | 11 (92%) |  |
|  |  |  |  | I (Mut) | 1 (8%) |  |
|  |  |  | 75 | N (WT) | 11 (92%) |  |
|  |  |  |  | E (Mut) | 1 (8%) |  |
|  |  |  | 76 | T (Mut) | 12 (100%) |  |
| pfmdr1 (n=12) | Morong, Bataan, Luzon | 1989-1991 | 86 | N (WT) | 1 (8%) | Chen et al., 2003 |
|  |  |  |  | Y (Mut) | 11 (92%) |  |
|  |  |  | 184 | Y (WT) | 11 (92%) |  |
|  |  |  |  | F (Mut) | 1 (8%) |  |
| pfcrt (n=8) | Morong, Bataan, Luzon | 1992-1993 | 72 | C (WT) | 8 (100%) |  |
|  |  |  | 74 | M (WT) | 8 (100%) |  |
|  |  |  | 75 | N (WT) | 8 (100%) |  |
|  |  |  | 76 | T (Mut) | 8 (100%) |  |
| pfmdr1 (n=8) | Morong, Bataan, Luzon | 1992-1993 | 86 | Y (Mut) | 8 (100%) | Chen et al., 2003 |
|  |  |  | 184 | Y (WT) | 8 (100%) |  |
| pfcrt (n=59) | Palawan island | 1997 | 72 | C (WT) | 33 (56%) | Sakihama et al., 2007 |
|  |  |  |  | S (Mut) | 26 (44%) |  |
|  |  |  | 73 | V (WT) | 59 (100%) |  |
|  |  |  | 74 | M (WT) | 59 (100%) |  |
|  |  |  | 75 | N (WT) | 59 (100%) |  |
|  |  |  | 76 | K (WT) | 33 (56%) |  |
|  |  |  |  | T (Mut) | 26 (44%) |  |
| pfcrt (n=30) | Tagum, Davao del Norte, Mindanao | 1999-2002 | 72 | C (WT) | 4 (13%) | Hatabu et al., 2009 |
|  |  |  |  | S (WT) | 26 (87%) |  |
|  |  |  | 73 | V (WT) | 30 (100%) |  |
|  |  |  | 74 | M (WT) | 26 (87%) |  |
|  |  |  |  | I (Mut) | 4 (13%) |  |
|  |  |  | 75 | N (WT) | 26 (87%) |  |
|  |  |  |  | I (Mut) | 4 (13%) |  |
|  |  |  | 76 | K (WT) | 26 (87%) |  |
|  |  |  |  | T (Mut) | 4 (13%) |  |
| pfmdr1 (n=30) | Tagum, Davao del Norte, Mindanao | 1999-2002 | 86 | N (WT) | 2 (7%) | Hatabu et al., 2009 |
|  |  |  |  | Y (Mut) | 28 (93%) |  |
| pfcrt (n=28) | Palawan Island | 2001-2005 | 72 | C (WT) | 20 (71%) | Hatabu et al., 2009 |
|  |  |  |  | S (Mut) | 8 (29%) |  |
|  |  |  | 73 | V (WT) | 28 (100%) |  |
|  |  |  | 74 | M (WT) | 27 (96%) |  |
|  |  |  |  | I (Mut) | 1 (4%) |  |
|  |  |  | 75 | N (WT) | 25 (89%) |  |
|  |  |  |  | E (Mut) | 1 (4%) |  |
|  |  |  |  | D (Mut) | 2 (7%) |  |
|  |  |  | 76 | K (WT) | 3 (11%) |  |
|  |  |  |  | T (Mut) | 25 (89%) |  |
| pfmdr1 (n=15) | Palawan Island | 2001-2005 | 86 | N (WT) | 10 (67%) | Hatabu et al., 2009 |
|  |  |  |  | Y (Mut) | 5 (33%) |  |
| pfcrt (n=17) | Kalinga | 2003-2005 | 72 | C (WT) | 3 (18%) | Hatabu et al., 2009 |
|  |  |  |  | S (Mut) | 14 (82%) |  |
|  |  |  | 73 | V (WT) | 17 (100%) |  |
|  |  |  | 74 | M (WT) | 17 (100%) |  |
|  |  |  | 75 | N (WT) | 17 (100%) |  |
|  |  |  | 76 | K (WT) | 1 (6%) |  |
|  |  |  |  | T (Mut) | 16 (94%) |  |
| pfmdr1 (n=8) | Kalinga | 2003-2005 | 86 | N (WT) | 5 (62%) | Hatabu et al., 2009 |
|  |  |  |  | Y (Mut) | 3 (38%) |  |
| pfcrt (n=38) | CARAGA region (Agusan del Sur, Agusan del Norte, Surigao del Sur | 2005-2006 | 72 | S (Mut) | 38 (100%) | Macalinao et al., 2020 |
|  |  |  | 76 | T (Mut) | 38 (100%) |  |
| pfdhfr (n=38) | CARAGA region (Agusan del Sur, Agusan del Norte, Surigao del Sur | 2005-2006 | 51 | N (WT) | 38 (100%) | Macalinao et al., 2020 |
|  |  |  | 59 | R (Mut) | 38 (100%) |  |
|  |  |  | 108 | N (Mut) | 38 (100%) |  |
| pfdhps (n=36 for 436, 437, 540; n=34 for 581, 613) | CARAGA region (Agusan del Sur, Agusan del Norte, Surigao del Sur | 2005-2006 | 436 | F (Mut) | 36 (100%) | Macalinao et al., 2020 |
|  |  |  | 437 | G (Mut) | 36 (100%) |  |
|  |  |  | 540 | K (WT) | 9 (25%) |  |
|  |  |  |  | E (Mut) | 27 (75%) |  |
|  |  |  | 581 | A (WT) | 34 (100%) |  |
|  |  |  | 613 | A (WT) | 34 (100%) |  |
| k13 (n=99) | Palawan Island | 2013 | 580, 539, 543, 493 | C (WT) | 99 (100%) | Menard et al., 2016 |
|  |  |  | 539 | R (WT) | 99 (100%) |  |
|  |  |  | 543 | I (WT) | 99 (100%) |  |
|  |  |  | 493 | Y (WT) | 99 (100%) |  |
| pfcrt (n=51) | Palawan Island | 2016 | See Table 1 | - | - | This study |
| pfmdr1 (n=90) | Palawan Island | 2016 | See Table 1 | - | - | This study |
| pfdhfr (n=123) | Palawan Island | 2016 | See Table 1 | - | - | This study |
| pfdhps (n=64) | Palawan Island | 2016 | See Table 1 | - | - | This study |
| k13 (n=57) | Palawan Island | 2016 | See Table 1 | - | - | This study |
| ^a^Unpublished data not included | |  |  |  |  |  |

| **Supplementary Table 3.** Data^a^ used for modelling temporal trends in *P. vivax* mutations | | | | | | |
| --- | --- | --- | --- | --- | --- | --- |
| **Genetic marker  (n of genotyped samples)** | **Study site** | **Date of collection** | **Amino acid position** | **Amino acid** | **Frequency (%)** | **Reference** |
| pvdhfr (n=15) | Agusan del Sur | 2002 | 33 | P (WT) | 15 (100%) | Auliff et al., 2006 |
|  |  |  | 50 | N (WT) | 15 (100%) |  |
|  |  |  | 57 | F (WT) | 15 (100%) |  |
|  |  |  | 58 | S (WT) | 5 (33%) |  |
|  |  |  |  | R (Mut) | 10 (67%) |  |
|  |  |  | 61 | T (WT) | 15 (100%) |  |
|  |  |  | 117 | S (WT) | 5 (33%) |  |
|  |  |  |  | N (Mut) | 10 (67%) |  |
|  |  |  | 173 | I (WT) | 15 (100%) |  |
| pvdhps (n=2) | Agusan del Sur | 2002 | 382 | S (WT) | 2 (100%) | Auliff et al., 2006 |
|  |  |  | 383 | G (Mut) | 2 (100%) |  |
|  |  |  | 512 | K (WT) | 2 (100%) |  |
|  |  |  | 553 | A (WT) | 2 (100%) |  |
|  |  |  | 585 | V (WT) | 2 (100%) |  |
| pvdhfr (n=87) | Palawan | 2009 | 13 | I (WT) | 87 (100%) | Bareng at al., 2018 |
|  |  |  | 33 | P (WT) | 86 (99%) |  |
|  |  |  |  | T (Mut) | 1 (1%) |  |
|  |  |  | 57 | F (WT) | 86 (99%) |  |
|  |  |  |  | L (Mut) | 1 (1%) |  |
|  |  |  | 58 | S (WT) | 17 (20%) |  |
|  |  |  |  | R (Mut) | 70 (80%) |  |
|  |  |  | 61 | T (WT) | 84 (97%) |  |
|  |  |  |  | M (Mut) | 2 (2%) |  |
|  |  |  |  | K (Mut) | 1 (1%) |  |
|  |  |  | 117 | S (WT) | 18 (21%) |  |
|  |  |  |  | N (Mut) | 67 (77%) |  |
|  |  |  |  | T (Mut) | 2 (2%) |  |
|  |  |  | 173 | I (WT) | 87 (100%) |  |
| pvdhps (n=100) | Palawan | 2009 | 382 | S (WT) | 100 (100%) | Bareng at al., 2018 |
|  |  |  | 383 | A (WT) | 28 (28%) |  |
|  |  |  |  | G (Mut) | 72 (72%) |  |
|  |  |  | 512 | K (WT) | 99 (99%) |  |
|  |  |  |  | N (Mut) | 1 (1%) |  |
|  |  |  | 553 | A (WT) | 100 (100%) |  |
|  |  |  | 585 | V (WT) | 95 (95%) |  |
|  |  |  |  | Y (Mut) | 2 (2%) |  |
|  |  |  |  | I (Mut) | 1 (1%) |  |
|  |  |  |  | S (Mut) | 1 (1%) |  |
| pvmdr1 (n=27) |  | 2016 | See table 1 | - | - | This study |
| pvdhfr (n=25) |  | 2016 | See table 1 | - | - | This study |
| pvdhps (n=18) |  | 2016 | See table 1 | - | - | This study |
| ^a^Unpublished data not included | | | | | | |

| **Supplementary Table 4.** | | | |
| --- | --- | --- | --- |
| Prevalence of haplotypes among sub-clinical and symptomatic samples | | | |
| **Marker** | **Haplotype** | **Sub-clinical; n/total (%)** | **Symptomatic; n/total (%)** |
| *pfmdr1* | NY (WT) | 42/90 (47) | 48/90 (53) |
| *pfcrt* | CVMNK (WT) | 24/51 (47) | 27/51 (53) |
| *pfdhfr* | NCS (WT) | 30/57 (24) | 27/57 (22) |
|  | NCN | 1/2 (1) | 1/2 (1) |
|  | NRN | 35/64 (29) | 29/64 (24) |
| *pfdhps* | FAKAA | 12/24 (19) | 12/24 (19) |
|  | FGKAA | 20/39 (31) | 19/39 (30) |
|  | FGEAA | 0/1 (0.0) | 1/1 (2) |
| *k13* | CRIY (WT) | 30/57 (53) | 27/57( 47) |
| *pvmdr1* | YL (WT) | 16/26 (59) | 10/26 (37) |
|  | FL | 0/1 (0) | 1/1 (4) |
| *pvdhfr* | IPFSTSI (WT) | 1/8 (4) | 7/8 (28) |
|  | IPFRTNI | 13/14 (52) | 1/14 (4) |
|  | IPFSTNI | 1/1 (4) | 0/1 (0) |
|  | IPLRMTI | 0/1 (0) | 1/1 (4) |
|  | LPFSTSI | 0/1 (0) | 1/1 (4) |
| *pvdhps* | SAKA (WT) | 6/11 (33) | 5/11 (28) |
|  | SGKA | 4/6 (22) | 2/6 (11) |
|  | SGKT | 0/1 (0) | 1/1 (6) |
